# Effects of adding *Sophora alopecuroides* to high concentrate diet on rumen fermentation parameters and microbial diversity of sheep

**DOI:** 10.3389/fvets.2023.1200272

**Published:** 2023-08-07

**Authors:** Yawen An, Hairong Wang, Zichao Zong, Zhixiong Gao, Caixia Shi, Shufang Li, Khas-Erdene

**Affiliations:** ^1^College of Animal Science, Inner Mongolia Agricultural University, Hohhot, Inner Mongolia, China; ^2^Key Laboratory of Animal Nutrition, Animal Nutrition and Feed Science, Hohhot, Inner Mongolia, China

**Keywords:** *Sophora alopecuroides*, high concentrate diet, rumen fermentation, rumen bacteria, rumen fungi, lambs

## Abstract

**Objective:**

The purpose of this study was to investigate the effects of different doses of Sophora alopecuroides (SA) on the rumen fermentation and microbial diversity of sheep.

**Methods:**

A total of 32 healthy Dumont crossbred male lambs weighing 25.73 ± 2.17 kg were randomly assigned to 4 treatment groups with 8 replicates each: a control group (CG) fed a basal diet with a concentrate-to-forage ratio of 7:3 and three experimental groups - the 0.1% group(TG1), 0.3% group (TG2), and 0.5% group (TG3), which were fed the same basal diet but supplemented with increasing doses of SA.

**Results:**

(1) Increasing the SA dose led to a significant linear increase (*p*-< 0.05) in acetate, propionate, butyrate, and total volatile fatty acid (TVFA) concentrations in the rumen, as well as a significant quadratic effect (*p*-< 0.05) on the propionate concentration. In contrast, there was a significant linear decrease (*p*-< 0.05) in the NH_3_-N concentration in the rumen. (2) At the level of rumen bacterial phyla, the abundance of Bacteroidetes in the rumen increased, and that of Firmicutes decreased (*p* = 0.08). At the genus level, the rumen abundances of Ruminococcus and Phocaeicola of sheep in the three experimental groups were significantly higher than in the control group (*p*-< 0.05), and the abundances of Clostridiales and Candidatus-Hepatincola were significantly increased in the 0.1% and 0.3% groups (*p* < 0.05). (3) Regarding rumen anaerobic fungi, the differences between the control group and experimental groups at the phylum level and genus level were not significant (*p* > 0.05), but the relative abundances of Neocallimastigomycota and Piromyces in the 0.1% group were significantly higher than that in the control group.

**Conclusion:**

SA addition to a high grain diet could increase the VFA concentration and pH in the sheep rumen, reduce the NH_3_-N concentration in the rumen and improve rumen fermentation function. Although there was no significant change in rumen bacterial or fungal diversity, SA addition increased the rumen abundances of *Bacteroidetes, Ruminococcus*, Phocaeicola, *Clostridiales, Neocallimastigomycota* and *Piromyces*, decreased the rumen abundance of *Firmicutes*, and had a positive effect on the rumen microbiota to improve sheep health.

## 1. Introduction

The necessary condition to improve ruminant performance and maintain their health of the organism is to maintain the balance of the rumen microecology, while the balance of the rumen microecosystem depends mainly on the diet. In recent years, in order to shorten the feeding cycle, accelerate the turnover and maximize the economic benefits, farmers have started to feed high concentrate diets to fattening sheep and high yielding cows. According to relevant reports, the fermentation of high grain diets in the rumen could easily cause the accumulation of short-chain volatile fatty acids in the rumen, resulting in a decrease in pH, which in turn could lead to changes in the rumen microbiota and subacute rumen acidosis (SARA) in severe cases (1, 2), thus affected the health of the organism. Although feeding antibiotics can provide some relief, they have been banned because of their ability to cause drug resistance in animals and residues in livestock products, which are harmful to human health (3). Therefore, finding substitutes for antibiotics to keep animals healthy has become a research hot spot in recent years.

*Sophora alopecuroides*, a Chinese herbal medicine, belongs to the genus Sophora of Leguminosae, which is mainly distributed in desert areas such as *Inner Mongolia* and *Ningxia* (4). It is rich in alkaloids, flavonoids, amino acids, fatty acids and other components (5). Among them, alkaloids are its main active ingredients, with anti-inflammatory and hypolipidemic, immunomodulatory effects (6). And it is mostly used for the treatment of gastrointestinal bleeding in monogastric animals (7). Some studies have found that alkaloids can effectively inhibit the growth of harmful bacteria, promote the proliferation of beneficial bacteria, improve the microflora in the intestinal tract of broilers, and promote the development of villus structure of small intestine in broilers, and improve the absorption function of the small intestine (8). Jia et al. (9) showed that 75-300 mg/kg SA alkaloids significantly improved the colitis induced by dextran sodium sulfate (DSS) in mice, inhibited expression of *IL-1β* and *TGF-β1* and upregulated *IL-10* expression. In addition, *in vivo* experiments using mice to establish models of *Helicobacter pylori* gastritis showed that total alkaloids of *Sophora alopecuroides* (TASA) combined with bismuth pectin or omeprazole could effectively reduce the expression levels of *IL-8*, *COX-2* and *NF-κB* in gastric mucosa infected with *Helicobacter pylori*, thus alleviating gastric mucosal inflammation (10). Li et al. (11) used 60 mg/kg TASAs for intraperitoneal perfusion in rats with ulcerative colitis (UC) and found that *S. alopecuroides* alkaloids may maintain a balance between pro-inflammatory and anti-inflammatory factors by increasing the levels of protective proteins, thereby alleviating the degree of inflammation as well as regulating the local immune response in the intestine. The results of previous studies revealed that the limited studies of SA and its extracts on gastrointestinal health were mostly focused on monogastric animals, while few studies on ruminants were reported. The group’s preliminary *in vitro* experiments showed that adding 0.1‒0.4% SA to the diet had positive effects on ruminal fermentation in sheep under high concentrate diets (12), but the specific effects, suitable additive amounts and mechanisms of action were unclear and needed further study.

Therefore, we hypothesized that the addition of different doses of SA to high concentrate diets for sheep would mitigate the hazards produced by high concentrate diets on the animal organism through the changing patterns of the rumen environment and microbial diversity (rumen bacteria and fungi). Our aim was to reveal the effect and mechanism of action of SA on rumen fermentation function, and further provide theoretical basis for the prevention and treatment of subacute rumen acidosis and the improvement of body health of ruminants under the condition of high concentrate by using SA as a feed additive.

## 2. Materials and methods

All experimental procedures were approved by Inner Mongolia Agricultural University Institutional Animal Care and Use Committee and conformed to national animal welfare regulations.

### 2.1. Experimental design and diet composition

In this experiment, thirty-two 3-months-old Dumont crossbred male lambs (25.73 ± 2.17 kg) were divided into 4 groups of 8 lambs each for immunization and deworming in a one-way completely randomized trial design. The test period was 15 days for the preliminary and 60 days for the main test, in which the control group (CG) was fed the basal diet; the TG1 group was fed the basal diet +0.1% SA; the TG2 group was fed the basal diet +0.3% SA; and the TG3 group was fed the basal diet +0.5% SA. The experimental diet was designed according to the table of common feed ingredients and nutritional value of Chinese sheep (NY/T 816–2004), and the diet formula and nutritional ingredients are shown in Table 1.

**Table 1:** Composition and nutrient levels of basal diets (DM basis).

Ingredients	Content/%	Nutrient levels	Content
Mixed hay	30.0	Metabolizable energy/(MJ·kg^−1^)	10.4
Corn	53.0	Crude protein/%	14.4
Soybean meal	14.9	Neutral detergent fiber/%	23.3
CaHPO_4_	0.10	Acid detergent fiber/%	15.0
NaCl	0.50	Nonstructural carbohydrate/%	49.9
Premix	0.50	Ca/%	0.70
NaHCO_3_	1.00	P/%	0.30
Total	100.0		

The premix provided the following per kg of diets: Ca 1520.0 mg, P 410.0 mg, Fe 25.0 mg, I 0.90 mg, Zn 35.0 mg, Co 0.10 mg, Cu 9.00 mg, Se 0.25 mg, Mn 19.5 mg, VE 15 IU, VD_3_ 1,000 IU, VA 3000 IU, nicotinic acid 60.0 mg. ME was a calculated value, while other nutrient levels were measured values.

Source and application method of SA: It was purchased from Yanchi, Ningxia. After being pulverized and sieved, it was mixed with the concentrate supplement at the corresponding addition ratio for feeding and the addition amount was selected based on the preliminary *in vitro* test conducted by our research group (12). The content of total alkaloids (main active substances) in SA is 5.75% by acidic dye spectrophotometry, and the recovery rate is 95.95%.

### 2.2. Feeding management

The feeding experiment was carried out in the teaching experimental pasture of Inner Mongolia Agricultural University. The sheep shed was thoroughly disinfected before the experiment. During the experiment, the experimental sheep were randomly assigned to 1.5 × 1 × 1 m iron cages for single cage feeding according to the principle of no significant difference in body weight between groups. The temperature and humidity of the sheep shed were kept at 15–20°C and 50–60%, respectively, with natural ventilation. The experimental sheep were fed twice a day at 08:00 and 18:00, with free access to feed and water.

### 2.3. Sample collection and determination index

#### 2.3.1. Rumen environment indicators

In the late stage of formal feeding, the rumen fluid was collected 0 h before intake, 2 h, 4 h, 6 h, 8 h and 10 h after intake by using oral sampling method, and the rumen fluid was filtered through four layers of gauze, measured ruminal pH of the rumen fluid, which was then put into a centrifuge tube and stored at −20°C for the determination of volatile fatty acid (VFA) and ammonia nitrogen (NH_3_-N) contents.

#### 2.3.2. Rumen microbial diversity

After the main test, five sheep of similar body weight in each group were selected for slaughter and collected the rumen chyme, divided in enzyme-free sterile centrifuge tubes and stored in liquid nitrogen. 16S rDNA and ITS rDNA high-throughput sequencing were analyzed by Shanghai Meiji Biological Technology Co., Ltd., and microbial diversity was detected by MiSeq PE300 sequencing platform to analyze the community species composition and abundance at the phylum and genus levels. We have uploaded the sequencing data of 16S rDNA and ITS rDNA to Sequence Read Archive (SRA) in NCBI (BioProject id: PRJNA963087).

### 2.4. Sequencing process

Firstly, sample DNA was extracted, specific primers with barcode in the assay region were synthesized, and the total DNA of the extracted samples was used as template to select specific primers for bacterial 16S rRNA and fungal ITS rRNA high variation regions for PCR amplification using an ABI GeneAmp® 9700 PCR instrument. The PCR products of the same samples were mixed and detected by 2% agarose gel electrophoresis was used to detect the PCR products, and the PCR products were recovered by cutting the gel using the AxyPrepDNA gel recovery kit (AXYGEN) and eluted with Tris–HCl; 2% agarose electrophoresis was used to detect the PCR products. The PCR products were detected and quantified by QuantiFluor™-ST Blue Fluorescence Quantification System (Promega), and then the samples were sequenced using Illumina MiSeq sequencing platform.

### 2.5. Bioinformatics analysis

The PE reads obtained from Miseq sequencing were firstly spliced according to the overlap relationship, while the raw data were filtered and chimeras were removed, etc. After that, the resulting sequences were OTU clustered using the software platform Usearch, and the sequences were clustered according to 97% similarity for non-repeated sequences (excluding single sequences) were OTU clustered, and sequences with 97% or more similarity to OTU representative sequences were selected to generate OTU tables. The representative sequences from OUT were compared with the microbial reference database, species annotation was performed, α-diversity was analyzed, and dilution curves and Veen plots were plotted using R language tools. The QIIME software was used to analyze the β-diversity and draw the principal coordinate analysis (PCoA) diagram. According to the results of taxonomic analysis, we can know what kind of microorganisms are contained in the samples and the sequence number of each microorganism in the sample, that is, the relative abundance of each microorganism. The data are analyzed and sorted out by Qiime platform.

### 2.6. Statistical analysis of data

All the recorded data were entered into an Excel table for preliminary collation. The MIXED PROC in SAS 9.2 statistical software was used for two-factor analysis of variance and regression analysis of rumen fermentation parameters. The two-factor analysis of variance included the dosage of SA, sampling time and interaction (SA × Time). The dose dependence of SA was analyzed by orthogonal polynomial analysis (Linear and Quadratic); The alpha diversity index of rumen bacteria and fungi was a two-tailed difference test using Student’ s *t*-test; the nonparametric Kruskal–Wallis *H*-test was used to compare the differences of rumen microbial flora in sheep. *p* < 0.05 indicated a significant difference or a significant regression relationship, and 0.05 < *p* < 0.10 indicated an upward or downward trend.

## 3. Results

### 3.1. Effect of SA on rumen fermentation parameters in sheep

As shown in Table 2, after different doses of SA were added, the rumen pH of TG3 group was significantly higher than that of CG and TG2 groups at 2 h of feeding (*p* < 0.01), the rumen pH of TG3 group was significantly higher than that of CG and TG1 groups at 4 h of feeding (*p* < 0.05), the rumen pH of TG1 and TG3 groups were significantly higher than that of CG and TG2 groups at 6 h of feeding (*p* < 0.05). Compared with the CG, ruminal NH_3_-N concentrations were significantly or highly significantly lower in the TG1, TG2, and TG3 groups at 4–8 h of feeding (*p* < 0.05 or *p* < 0.01), and there were no significant differences in NH_3_-N concentrations between the groups at the other time points (*p* > 0.05). With the increase of the dosage of SA, the concentration of NH_3_-N in the rumen presented a linear significant decrease (*p* < 0.05), and the quadratic curve showed a trend of decrease (*p* = 0.07). With the prolongation of sampling time, the concentration of NH_3_-N in the rumen presented a change rule of increase first and then decrease, and reached the peaked at 2 h.

**Table 2:** Effects of SA in high concentrate diet on rumen fermentation of sheep.

Time	Group (SA)	pH	NH_3_-N (mmol/L)	Acetate (mmol/L)	Propionate (mmol/L)	Butyrate (mmol/L)	TVFA (mmol/L)	Acetate/propionate
0 h	CG	6.99	27.63	25.93ab	12.96ab	3.59ab	42.49a	2.01ab
	TG1	6.95	20.64	27.95a	13.20a	3.50ab	44.66a	2.13a
	TG2	6.82	22.56	20.22c	11.16b	3.24b	34.62c	1.81b
	TG3	6.99	23.12	27.21a	13.40a	3.79a	44.4a	2.03ab
2 h	CG	5.41	36.77	47.16c	18.91b	5.04c	71.10c	2.51b
	TG1	5.67	33.08	60.6a	21.92a	6.13ab	88.65a	2.77a
	TG2	5.48	36.48	64.34a	22.61a	6.56a	93.51a	2.85a
	TG3	5.99	30.55	63.68a	22.10a	5.84b	91.62a	2.88a
4 h	CG	5.52	31.98	43.78c	24.79	6.30	74.87bc	1.77c
	TG1	5.51	23.12	52.92a	24.33	6.35	83.61a	2.18a
	TG2	5.72	26.30	47.90b	24.41	6.50	78.81b	1.96b
	TG3	6.03	22.74	47.95b	23.59	6.15	77.68b	2.03ab
6 h	CG	5.71	32.52	32.48b	17.73bc	5.22b	55.43bc	1.84b
	TG1	6.32	20.53	33.97b	18.34b	4.96bc	57.27b	1.85b
	TG2	5.72	24.58	32.65b	18.12b	6.45a	57.22b	1.79b
	TG3	6.27	21.62	42.67a	21.36a	5.67ab	69.70a	2.00a
8 h	CG	6.68	35.48	32.34b	16.73b	4.61b	53.68b	1.93b
	TG1	6.61	17.44	34.39ab	16.48b	4.45bc	55.31b	2.08a
	TG2	6.17	20.57	25.24c	13.93c	4.04c	43.21c	1.81c
	TG3	6.52	20.18	40.20a	21.92a	6.71a	68.83a	1.83c
10 h	CG	6.55	24.73	31.91	14.95	4.12	50.98	2.13
	TG1	6.35	21.47	33.35	15.64	4.27	53.26	2.13
	TG2	6.36	20.22	31.79	15.02	4.01	50.82	2.12
	TG3	6.40	21.98	31.47	15.32	4.34	51.13	2.05
SEM		0.09	2.24	0.89	0.38	0.33	1.18	0.04
**Main effects**								
SA	CG	6.14	31.52a	35.60c	17.68c	4.82c	58.09c	2.03c
	TG1	6.23	22.71c	40.53a	18.32b	4.94bc	63.79a	2.19a
	TG2	6.04	25.12c	37.02bc	17.54c	5.13b	59.70bc	2.06bc
	TG3	6.37	23.37c	42.20a	19.62a	5.42a	67.23a	2.14a
Time	0 h	6.94a	23.49c	25.33d	12.68e	3.53e	41.54e	1.99c
	2 h	5.64c	34.22a	58.95a	21.38b	5.89b	86.22a	2.75a
	4 h	5.70c	26.04c	48.14b	24.28a	6.32a	78.74b	1.99c
	6 h	6.01bc	24.81c	35.44c	18.89c	5.58b	59.91c	1.87d
	8 h	6.49b	23.42c	33.04c	17.27 cd	4.95c	55.26 cd	1.91 cd
	10 h	6.41b	22.10c	32.13c	15.23d	4.19d	51.55d	2.11b
*p*-value	SA	0.11	<0.01	<0.01	<0.01	<0.01	<0.01	<0.01
	Time	<0.01	<0.01	<0.01	<0.01	<0.01	<0.01	<0.01
	SA × Time	0.75	0.91	<0.01	<0.01	<0.01	<0.01	0.01
	Linear	0.24	0.01	<0.01	<0.01	<0.01	<0.01	0.35
	Quadratic	0.13	0.07	0.27	<0.01	0.74	0.07	0.68

Data marked without the same lowercase letters in each column indicated significant differences at *p* < 0.05, interphase lowercase letters indicated highly significant differences at *p* < 0.01. The same as below.

At 0 h before feeding, the concentrations of acetate and TVFA in TG2 group were significantly lower than those in other groups (*p <* 0.01), the concentration of propionate and the ratio of acetate to propionate in TG2 group was significantly lower than that in TG1 group (*p <* 0.05), the concentration of butyrate in TG3 group was significantly higher than that in TG2 group (*p <* 0.05). At 2 h after feeding, the concentrations of acetate, propionate, butyrate and TVFA in rumen of the three experimental groups were significantly or extremely significantly higher than those of the control group (*p <* 0.05 or *p <* 0.01). At 4 h after feeding, the concentrations of rumen acetate, TVFA and the ratio of acetate to propionate in TG1, TG2 and TG3 groups were significantly or extremely significantly higher than CG group (*p <* 0.05 or *p <* 0.01), and TG1 group was the highest. At 6 h after feeding, the concentrations of acetate, propionate and TVFA in the rumen of TG3 group were significantly or extremely significantly higher than those of the other three groups (*p <* 0.05 or *p <* 0.01), while the concentrations of butyrate in the rumen of TG2 group was significantly or extremely significantly higher than the CG and TG1 group (*p <* 0.05 or *p <* 0.01). At 8 h after feeding, the rumen acetate concentration in TG3 group was significantly higher than that in the control group (*p <* 0.05), while that in TG1 and TG3 groups were significantly higher than that in TG2 group (*p <* 0.01). The rumen propionate, butyrate and TVFA concentrations in TG3 group were significantly or extremely higher than those in the other three groups (*p <* 0.05 or *p <* 0.01). The concentration of butyrate in TG2 group was significantly lower than that in the control group (*p <* 0.05), and the ratio of acetate to propionate in TG1 group was significantly or extremely significantly higher than that in the other three groups (*p <* 0.05 or *p <* 0.01). At 10 h after feeding, there were no significant difference in each index all the groups (*p* > 0.05).

With the increase of the dosage of SA, the concentrations of acetate, propionate, butyrate and TVFA in rumen all increased linearly (*p* < 0.05), the concentration of propionate in rumen showed a significant quadratic curve effect (*p* < 0.05), and the concentration of butyrate in rumen showed a quadratic curve effect (*p* = 0.07). The sampling time is closely related to various parameters of rumen fermentation. With the extension of sampling time, the concentrations of acetate, propionate, butyrate and TVFA in rumen all showed a trend of increasing first and then decreasing, and the concentrations of acetate and TVFA reached the peak at 2 h, while those of propionate and butyrate reached the peak at 4 h.

### 3.2. Effect of SA on diversity of rumen bacteria in sheep

#### 3.2.1. Analysis of alpha diversity of rumen bacteria

Sobs refers to the number of OTU actually observed, Shannon and Simpson mainly reflect species diversity, and Ace and Chao measure species richness. It can be seen from Table 3 that Shannon was TG1 > TG2 > CG > TG3, and Simpson was TG3 > TG1 > CG = TG2, but the difference was not significant (*p* > 0.05), which showed that the diversity of rumen bacteria with different doses of SA in high-concentrate diet was similar. Sobs, Ace and Chao in the three experimental groups were all higher than those in the control group, and there was no significant difference among the groups (*p* > 0.05), indicating that the species richness of the four groups were basically the same.

**Table 3:** Difference test of alpha diversity index of rumen flora among groups.

Group	Sobs	Shannon	Simpson	Ace	Chao
CG	145.00	3.22	0.08	155.14	155.41
TG1	154.75	3.29	0.09	163.49	163.79
TG2	149.25	3.25	0.08	157.88	163.67
TG3	147.25	3.10	0.12	155.86	158.48
SEM	6.27	0.11	0.02	6.70	8.02
*p*-value (CG-TG1)	0.25	1.00	0.67	0.47	0.67
*p*-value (CG-TG2)	0.47	0.67	0.67	0.89	0.89
*p*-value (CG-TG3)	1.00	0.47	0.31	0.89	1.00
*p*-value (TG2-TG3)	0.77	0.31	0.31	0.89	0.89
*p*-value (TG2-TG1)	0.67	0.89	0.89	0.89	0.89
*p*-value (TG3-TG1)	0.31	0.31	0.31	0.31	0.67

#### 3.2.2. Composition of rumen bacteria

**Figure 1:**
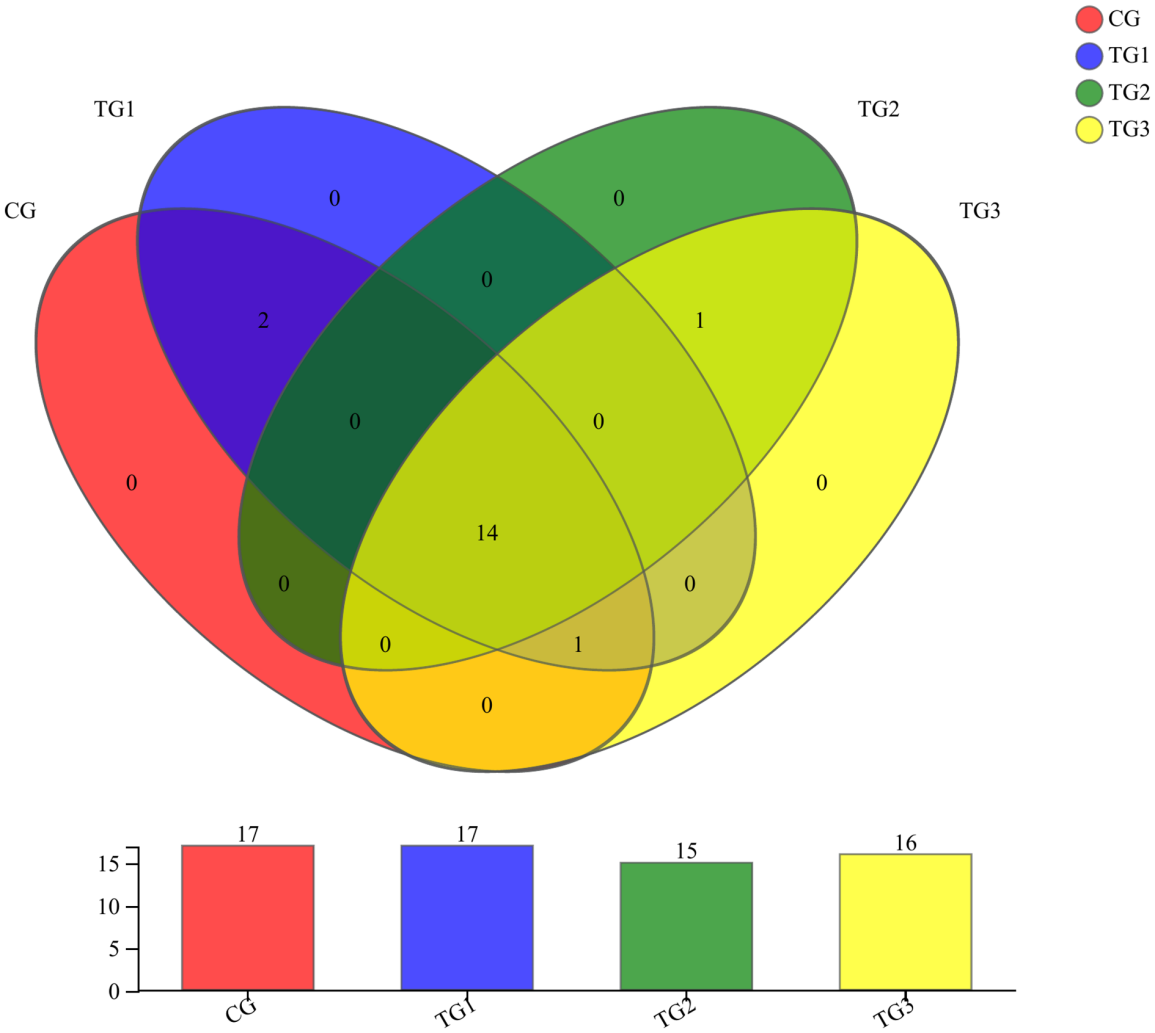
Venn diagram of rumen bacteria at phylum levels. Different colors represent different groups, numbers in overlapping parts represent the number of species common to the plurality of groups, and numbers in non-overlapping parts represent the number of species specific to the corresponding group.

As shown in Figure 1 and Table 4 below, at the phylum level, there were 17 phyla in CG and TG1 groups, 15 phyla in TG2 group and 16 phyla in TG3 group, of which 14 phyla were shared by the control group and the experimental group. Mainly *Actinobacteria*, *Firmicutes*, *Chloroflexi*, etc. The common phylum of CG group and TG1 group were *Armatimonadetes* and *Fusobacteria*, the common phylum of CG group and TG1 and TG3 group were *Absconditabacteria*, and the common phylum of TG2 and TG3 group were *Lentisphaerae*.

**Table 4:** Classification of rumen bacteria at phylum level.

Group	Bacteria phylum
CG&TG1&TG2&TG3	*Actinobacteria, Elusimicrobia, Tenericutes, Firmicutes, Cyanobacteria, unclassified-k-norank, Chloroflexi, Bacteroidetes, Verrucomicrobia, Spirochaetae, Saccharibacteria, Ynergistetes, Proteobacteria, Fibrobacteres*
CG&TG1	*Armatimonadetes fusobacteria*
CG&TG1&TG3	*Absconditabacteria*
TG2&TG3	*Lentisphaerae*

**Figure 2:**
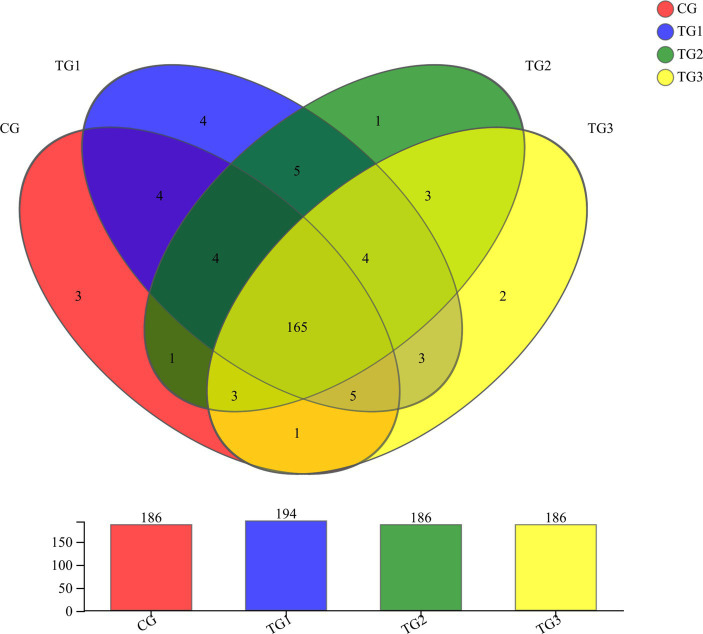
Venn diagram of rumen bacterial at genus levels (Figure 1).

As shown in Figure 2 and Table 5 below, at genus level, there were 186 genera in CG, TG2 and TG3 groups. 194 genera in TG1 group, and 165 genera in common in control group and the experimental groups. Mainly include *Paraprevotella*, *Papillibacter*, *Terrisporobacter*, *Succiniclasticum*, etc. The endemic genera in CG group was *Oscillospira*, *Marinilabiaceae* and *Fibrobacteriaceae*. The specific genera of TG1 were *Syntrophomonas*, *Rickettsiales*, *Jeotgalicoccus* and *Eubacterium*; the specific genus in TG2 group was *Staphylococcus*. The genera endemic to TG3 group were *Pseudoscardovia* and *Dialister*. The common genera in TG1, TG2 and TG3 groups were *Hydrogenoanaerobacterium*, *Ruminiclostridium*, *Erysipelotrichaceae* and *Mitsuokella*.

**Table 5:** Classification of rumen bacteria at genus level.

Group	Bacteria genus
CG&TG1&TG2&TG3	*Paraprevotella, Papillibacter, Terrisporobacter, Fretibacterium, Succiniclasticum, Fibrobacter, Roseburia, Desulfovibrio, Bifidobacterium, Ruminococcus, unclassified-Firmicutes, Oribacterium, Lachnoclostridium*
CG	*Oscillospira, Marinilabiaceae, Fibrobacteraceae*
TG1	*Syntrophomonas, Rickettsiales, Eubacterium, Jeotgalicoccus*
TG2	*Staphylococcus*
TG3	*Dialister, Pseudoscardovia*
TG1&TG2&TG3	*Hydrogenoanaerobacterium, Ruminiclostridium, Erysipelotrichaceae, Mitsuokella*

#### 3.2.3. Species richness at the level of rumen bacterial phylum and genus

**Figure 3:**
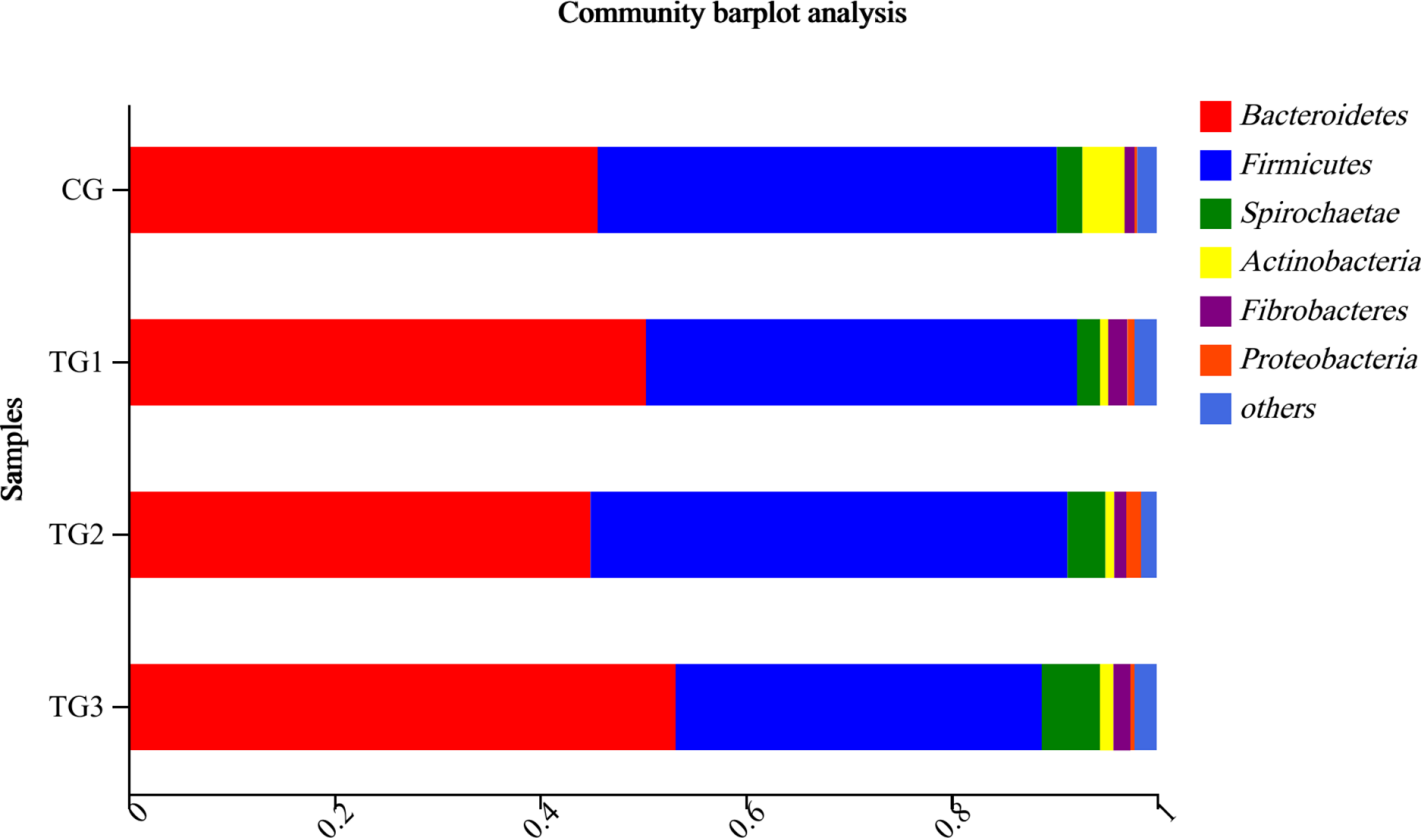
Horizontal column chart of rumen bacteria at phylum levels. The ordinate indicates the grouping, the abscissa is the proportion of species in the group, different colors represent different species, and the length represents the proportion of the species.

As shown in Table 6 and Figure 3, *Bacteroidetes* and *Firmicutes* were the dominant phyla in each group. Compared to the control group, *Bacteroidetes* had a tendency to increase the percentage of TG3 group (*p* = 0.08), while *Firmicutes* had a tendency to decrease in the TG3 group (*p* = 0.07). There were no significant differences in other phyla between the groups (*p* > 0.05).

**Table 6:** Species abundance differences of rumen bacteria at phylum level/%.

Phylum name	*Bacteroidetes*	*Firmicutes*	*Spirochaetae*	*Actinobacteria*	*Fibrobacteres*	*Proteobacteria*	*Synergistetes*	*Saccharibacteria*
CG	45.91 ± 10.18	44.66 ± 6.85	2.38 ± 2.35	3.93 ± 4.20	1.07 ± 1.58	0.24 ± 0.15	0.47 ± 0.38	0.51 ± 0.67
TG1	45.91 ± 5.48	41.91 ± 3.99	2.27 ± 0.67	0.86 ± 0.49	1.80 ± 1.01	0.71 ± 0.36	0.80 ± 1.07	0.35 ± 0.22
TG2	44.97 ± 3.63	46.38 ± 3.47	3.60 ± 2.58	0.85 ± 0.40	1.16 ± 1.51	1.44 ± 2.12	0.64 ± 0.49	0.25 ± 0.18
TG3	53.28 ± 2.86	35.66 ± 4.86	5.63 ± 3.66	1.23 ± 1.75	1.67 ± 0.77	0.40 ± 0.22	0.42 ± 0.59	0.57 ± 0.35
*P*-value	0.08	0.07	0.42	0.62	0.85	0.22	0.89	0.52

**Figure 4:**
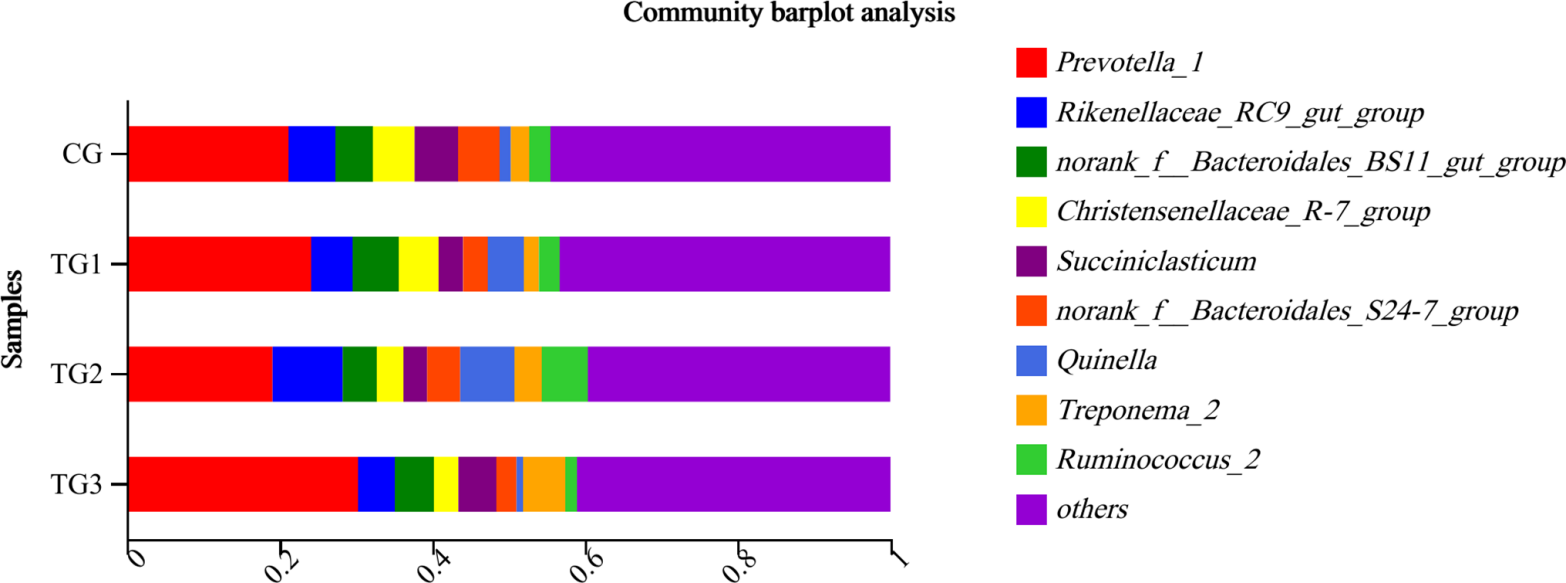
Horizontal column chart of rumen bacteria at genus levels (Figure 3).

As shown in Figure 4 and Table 7 below, rumen bacteria were subjected to taxonomic statistics at the genus level, and 7 kinds of rumen bacteria with significant difference or difference trend were obtained. The proportion of *Ruminococcus* and *Phocaeicola* in the experimental groups were significantly higher than that in CG group, especially in TG1 group. The proportion of *Sphaerochaeta*, *Clostridiales* and *Candidatus-Hepatincola* in TG1 and TG2 groups were higher than that in CG group (*p* = 0.09 or *p <* 0.05). The proportion of *Eubacterium* in TG2 group was lower than that in CG group (*p* = 0.07). The percentages of *Asterolemia* in TG1, TG2 and TG3 groups were lower than that in CG group (*p* = 0.09).

**Table 7:** Species abundance differences of rumen bacteria at genus level/%.

Genus name	*Ruminococcus*	*Phocaeicola*	*Sphaerochaeta*	*Clostridiales*	*Eubacterium*	*Candidatus-Hepatincola*	*Asteroleplasma*
CG	0.57 ± 0.32b	0.08 ± 0.01b	0.04 ± 0.05	0.05 ± 0.08b	0.05 ± 0.02	0.02 ± 0.03b	0.05 ± 0.08
TG1	1.35 ± 0.17a	0.35 ± 0.15a	0.20 ± 0.09	0.09 ± 0.05a	0.05 ± 0.04	0.07 ± 0.03a	0.02 ± 0.03
TG2	1.15 ± 0.47a	0.24 ± 0.11b	0.09 ± 0.06	0.10 ± 0.02a	0.03 ± 0.01	0.04 ± 0.07a	0.03 ± 0.01
TG3	1.63 ± 1.16a	0.21 ± 0.11b	0.05 ± 0.02	0.03 ± 0.01b	0.05 ± 0.02	0.00 ± 0.00b	0.00 ± 0.00
*P*-value	0.04	0.03	0.09	0.01	0.07	0.02	0.09

### 3.3. Effect of adding SA in high-concentrate diet on the diversity of rumen fungi in sheep

#### 3.3.1. Analysis of alpha diversity of rumen fungi

As shown in Table 8, Simpson ranked in the order of TG1 > TG2 > TG3 > CG, but the differences were not significant (*p* > 0.05). The Sobs, Shanon, Ace and Chao indexes of the control group were slightly higher than those of the three experimental groups in numerical value, but the differences were not significant among different groups. These results indicated that the diversity and abundance of rumen fungi when different doses of SA were added into high concentrate diets were similar.

**Table 8:** Difference test of alpha diversity index of rumen fungi among groups.

Group	Sobs	Shannon	Simpson	Ace	Chao
CG	52.75	1.39	0.48	60.37	55.44
TG1	43.00	0.49	0.75	59.75	52.92
TG2	33.75	0.81	0.65	40.31	40.50
TG3	30.25	1.19	0.52	53.60	39.52
SEM	8.52	0.18	0.07	8.34	8.27
*P*-value(CG-TG1)	0.67	0.31	0.19	0.67	0.47
*P*-value(CG-TG2)	0.31	0.67	0.47	0.31	0.31
*P*-value(CG-TG3)	0.19	0.89	1.00	1.00	0.67
*P*-value(TG1-TG2)	0.88	0.47	0.67	0.47	0.67
*P*-value(TG1-TG3)	1.00	0.47	0.31	0.89	0.89
*P*-value(TG2-TG3)	0.67	1.00	0.67	0.31	1.00

#### 3.3.2. Rumen fungal composition

**Figure 5:**
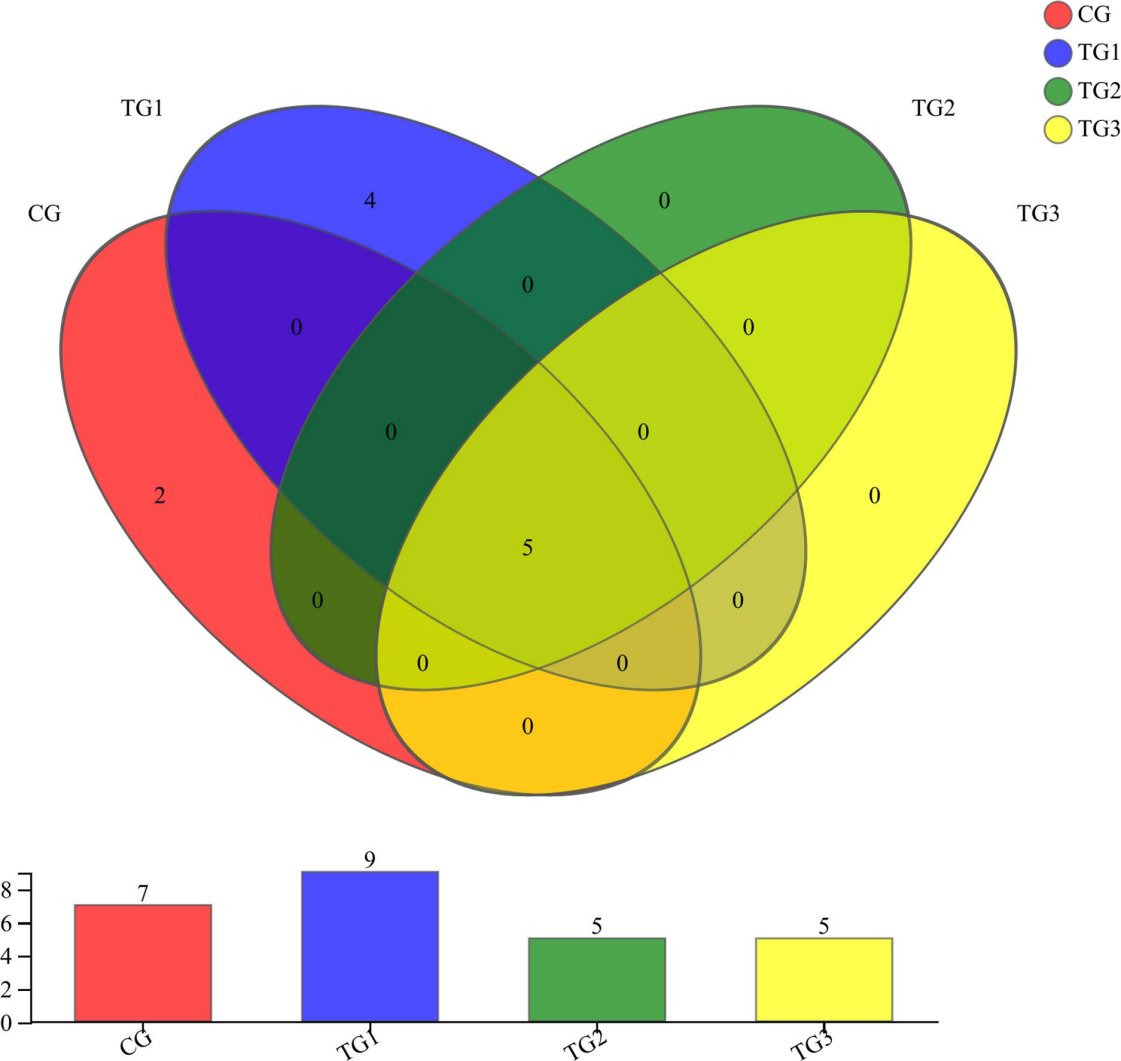
Venn diagram of rumen anaerobic fungi at phylum levels (Figure 1).

As shown in Figure 5 and Table 9 below, at the phylum level, CG group had 7 phyla, TG1 group had 9 phyla, TG2 and TG3 groups each had 5 phyla, among which 5 phyla were shared by the control group and the three experimental groups. They were *Ascomycota*, *Neocallimastigomycota*, etc. The unique anaerobic fungi in CG group were *Cercozoa* and *Glomeromycota*, and the unique anaerobic fungi in TG1 group were *Chytridiomycota*, *Mucoromycota*, etc.

**Table 9:** Classification of rumen anaerobic fungi at phylum level.

Group	Phylum fungi
CG&TG1&TG2&TG3	*Ascomycota, Neocallimastigomycota, Mortierellomycota, unclassified-Fungi, Basidiomycota*
CG	*Cercozoa, Glomeromycota*
TG1	*Chytridiomycota, Mucoromycota, Rozellomycota, Olpidiomycota*

**Figure 6:**
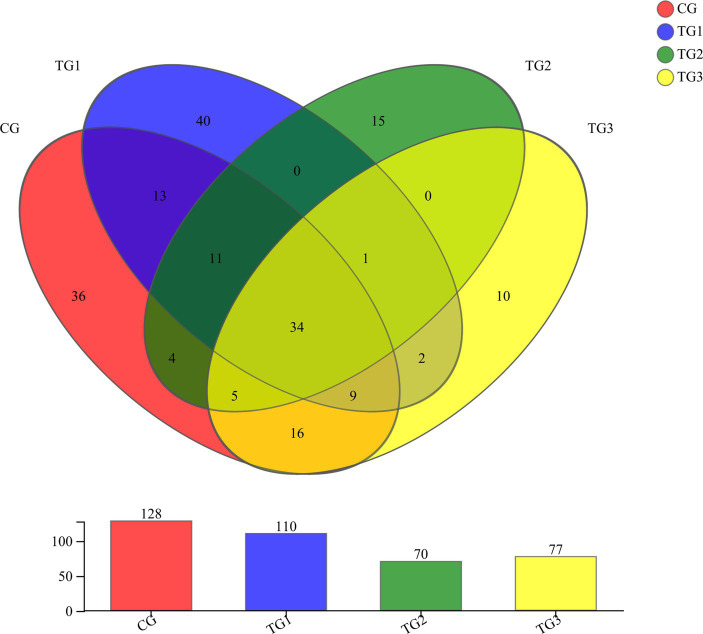
Venn diagram of rumen anaerobic fungi at genus levels (Figure 1).

As shown in Figure 6 and Table 10 below, at the genus level, there were 128 genera in the CG group, 110 genera in the TG1 group, 70 genera in the TG2 group, 77 genera in the TG3 group, and 34 genera common to the control and experimental groups, mainly including *Unclassified-Chaetomiaceae*, *Filobasidium*, *Unclassified-Didymellaceae*, etc. There were 40 species of fungal genera endemic to TG1 group, mainly *Ramularia*, *Geminibasidium*, *Cordyceps*, etc. There were 15 fungal genera endemic to TG2 group, mainly *Phialemonium*, *Cotylidia*, *Thanatephorus*, etc. There were 10 endemic fungal genera in TG3 group, mainly *Mrakia*, *Uwebraunia*, *Lectera*, etc. The fungal genera shared by TG1, TG2 and TG3 groups were *Sarocladium*.

**Table 10:** Classification of rumen anaerobic fungi at genus level.

Group	Fungi genus
CG&TG1&TG2&TG3	*unclassified-Chaetomiaceae, Filobasidium, unclassified-Didymellaceae, unclassified-Basidiomycota, Microascus, Oidiodendron, Cladosporium, unclassified-Microascaceae, Mycosphaerella, Acrostalagmus, Piromyces, Fusarium, unclassified-Fungi*
CG	*unclassified-Ustilaginacea, Selenophoma, Setophaeosphaeria, Coprinopsis, Hygrocybe, Myrmecridium, Debaryomyces, Cylindrocladiella, Trichomerium, Pseudorobillarda, unclassified-Pyronemataceae, unclassified-Chionosphaeraceae, Pseudaleuria, unclassified-Apiosporaceae, Chrysosporium, unclassified-Chaetothyriales, Clavulinopsis, Chloridium, unclassified-Bionectriaceae*
TG1	*Ramularia, Geminibasidium, Cordyceps, Cystofilobasidium, Wrightoporia, Paraboeremia, unclassified-Chytridiomycota, Preussia, Beauveria, Stachybotrys, unclassified-Thelephoraceae, unclassified-Aspergillaceae, Sclerostagonospora, unclassified-Xylariales, Olypocladium, Ceratobasidium, Podospora, unclassified-Olpidiales, Russula, unclassified-Pleosporaceae, Archaeorhizomyces, Serendipita, Bifiguratus, unclassified-Helicobasidiaceae, unclassified-Chaetosphaeriaceae, unclassified-Sordariales*
TG2	*Phialemonium, Phialocephala, Cotylidia, Thanatephorus, Sporobolomyces, Guehomyces, Pseudogymnoascus, Rhodotorula, unclassified-Cystofilobasidiales, Engyodontium, unclassified-Cephalothecaceae, Arachnomyces, Thermomyces, Malassezia*
TG3	*Mrakia, Uwebraunia, Lectera, Castanediella, Clitopilus, Corynespora, Orpinomyces, Xerochrysium, Dissoconium, Monodictys*
TG1&TG2&TG3	*Sarocladium*

#### 3.3.3. Species richness at the level of rumen fungal phylum and genus

**Figure 7:**
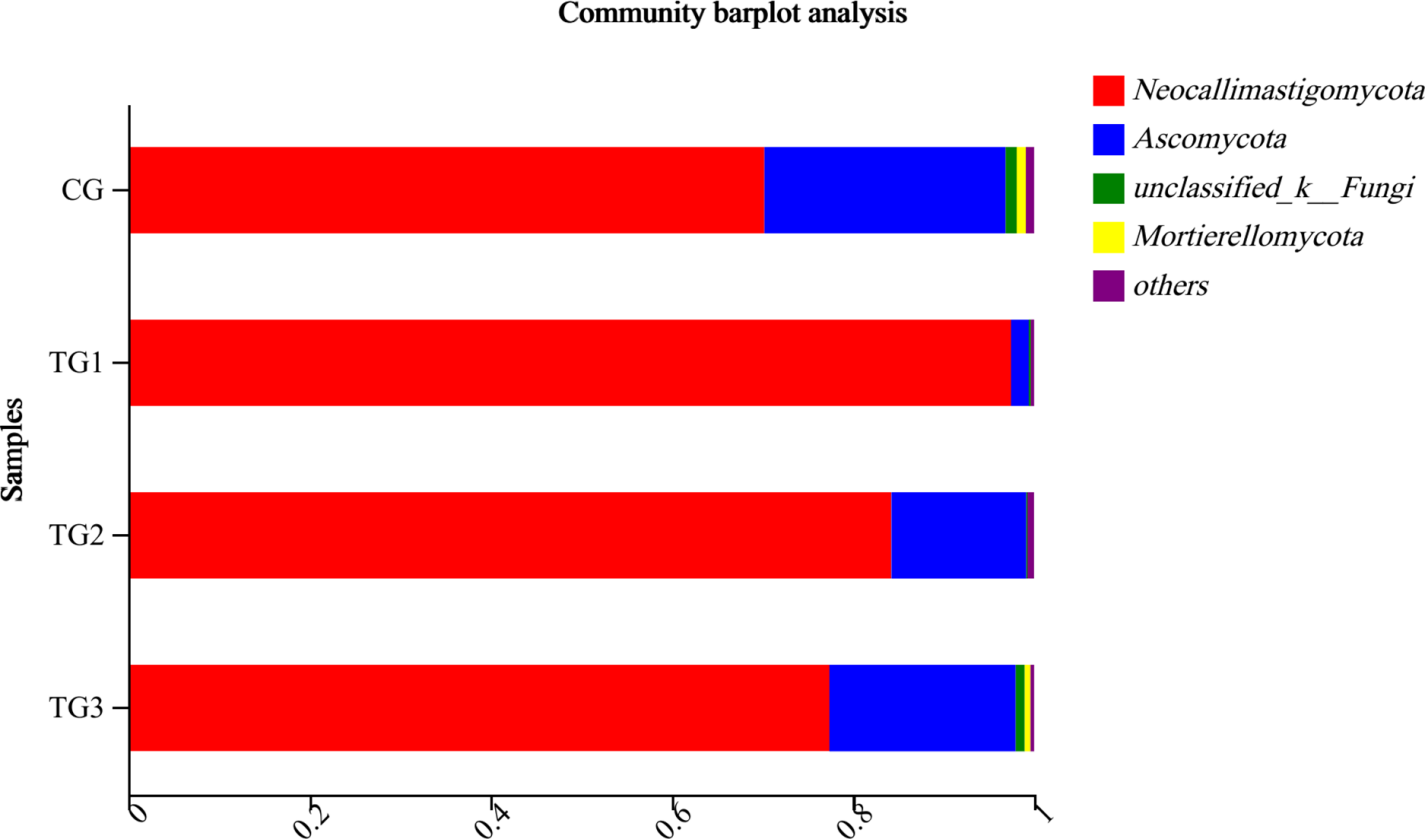
Horizontal column chart of rumen fungi at phylum levels (Figure 3).

As can be seen from Table 11 and its Figure 7, the control and experimental groups of *Neocallimastigomycota*, *Ascomycota*, *Unclassified-Fungi*, *Basidiomycota*, and *Mortierellomycota* were not significantly different at the phylum level (*p* > 0.05).

**Table 11:** Species abundance differences of rumen fungi at phylum level/%.

Phylum name	*Neocallimastigomycota*	*Ascomycota*	*unclassified-Fungi*	*Basidiomycota*	*Mortierellomycota*
CG	70.22 ± 37.62	26.62 ± 33.58	1.24 ± 1.62	0.87 ± 0.95	1.02 ± 1.58
TG1	97.49 ± 3.06	1.94 ± 2.09	0.20 ± 0.37	0.35 ± 0.58	0.01 ± 0.01
TG2	84.25 ± 16.13	14.89 ± 15.03	0.13 ± 0.11	0.72 ± 1.29	0.00 ± 0.00
TG3	77.41 ± 43.57	20.57 ± 39.65	0.99 ± 1.87	0.40 ± 0.77	0.64 ± 1.28
*p*-value	0.46	0.48	0.19	0.58	0.23

**Figure 8:**
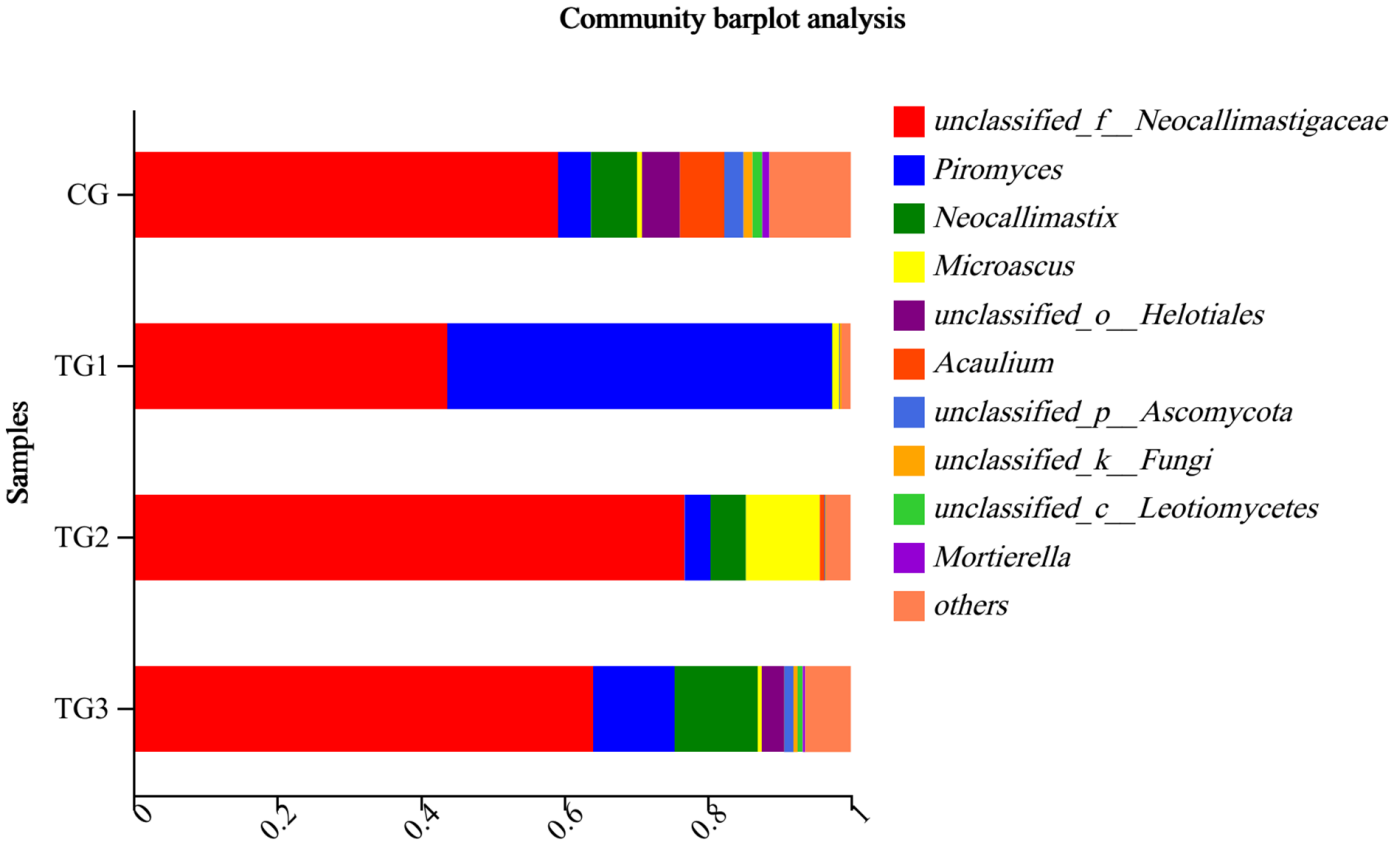
Horizontal column chart of rumen fungi at genus levels (Figure 3).

As shown in Table 12 and Figure 8, the control and experimental groups of *Unclassified-Neocallimastigaceae*, *Piromyces*, *Neocallimastix* were not significantly different at the genus level (*p* > 0.05).

**Table 12:** Species abundance differences of rumen fungi at genus level/%.

Genus name	*unclassified-Neocallimastigaceae*	*Piromyces*	*Neocallimastix*	*Microascus*	*unclassified-Helotiale*	*Acaulium*	*unclassified-Ascomycota*	*unclassified-Fungi*
CG	58.81 ± 32.23	4.40 ± 3.19	7.02 ± 13.98	0.68 ± 1.09	5.41 ± 10.82	5.74 ± 11.31	2.83 ± 5.43	1.24 ± 1.62
TG1	41.21 ± 41.91	56.23 ± 41.61	0.05 ± 0.10	0.93 ± 0.89	0.00 ± 0.00	0.04 ± 0.02	0.09 ± 0.17	0.20 ± 0.37
TG2	74.74 ± 25.70	3.53 ± 4.72	5.98 ± 11.96	10.76 ± 10.58	0.01 ± 0.01	0.74 ± 1.31	0.11 ± 0.13	0.13 ± 0.11
TG3	56.25 ± 39.14	11.58 ± 16.88	9.58 ± 19.12	0.66 ± 0.51	5.36 ± 10.72	0.13 ± 0.24	2.28 ± 4.53	0.99 ± 1.87
*p*-value	0.74	0.26	0.97	0.11	0.76	0.41	0.47	0.19

## 4. Discussion

### 4.1. Effect of adding SA in high concentrate diet on rumen fermentation parameters of sheep

Rumen pH is an important indicator to maintain normal rumen fermentation of animals. The normal pH range of the rumen is 6.0–7.5. The rumen pH (5.2–5.6) exceeding 3 h is often recognized as the cause of SARA, and acute acidosis occurs when pH is below 5.0 (13). The pH value is affected by the feeding method, concentrate-to-forage ratio and dry matter level. Many studies have found that frequent intake of high-grain diets by animals will lead to a decrease in the pH of the rumen. When the pH falls to 6.0, a slight decrease in the fiber decomposition rate will be presented, but the number of fiber decomposition bacteria is generally not affected. When it falls to 5.5 or 5.0, it will lead to a decrease in both fiber decomposition rate and fiber decomposition bacteria, and may even completely inhibit fiber digestion, thereby changing the rumen fermentation function (14). This study showed that the rumen pH of the control group was 5.4, 5.5, and 5.7 when the animals were fed for 2 h, 4 h, and 6 h, respectively, indicating that there was a trend of SARA in the control group. While the rumen pH of the TG3 group was about 6.0 during this time, significantly higher than that of the control group, and indicating that SA added in the high-concentrate diet was conducive to improving the rumen pH under high concentrate diets, and might play an important role in the utilization of cellulose by microorganisms to change rumen fermentation functions. In addition, the pH values in the rumen of the other three experimental groups were relatively stable, which had a positive effect on the maintenance of the homeostasis of the rumen environment, which was similar to the result of Gu et al. (15) who added Chinese herbal medicine additives into the diet to improve the pH of rumen and maintain the stability of the intragastric environment.

Ammonia nitrogen concentration is a dynamic indicator of the degradation of ammonia and nitrogen sources by rumen microorganisms, which can reflect the rate of microbial degradation and synthesis of nitrogen-containing substances as well as the utilization of ammonia nitrogen by microorganisms (16). Rumen microorganisms can utilize ammoniacal nitrogen to synthesize protein, and the appropriate concentration of NH_3_-N is beneficial to the synthesis of bacterial protein for use by the animal organism. Pablo et al. (17) found that with the increase of the dosage of the composite herbal medicine, the concentrations of NH_3_-N and TVFA in the rumen of lambs tended to increase first and then decrease, which might be related to the synergistic effect of the active components in the composite herbal medicine. However, Vera et al. (18) found that both pine bark extract and mignonette extract could reduce the concentration of NH_3_-N in the rumen, which might be due to the fact that the polyphenol tannin in pine bark and mignonette extract could inhibit the activities of protease and microbial deaminase, and thus forming tannin complex to reduce NH_3_-N production at rumen pH = 6–7. The results of this experiment showed that the ammonia nitrogen concentrations in the three experimental groups were significantly reduced 4–8 h after intake, and the NH_3_-N concentration in TG1 group was the lowest. The previous weight gain results of our research group also showed that the average daily weight gain and F/G of the 0.1% SA group were significantly increased (19), indicating that the addition of different doses of SA in the high-precision diet might improve the utilization rate of ammonia nitrogen by rumen microorganisms, promote rumen fermentation and play a role in promoting growth, and the effect of 0.1% addition was better.

VFA produced by ruminant fermentation mainly include acetate, propionate and butyrate, etc. They are the main source of energy for ruminant growth, production and reproduction, and can provide 70 to 80% of total energy for animals to use. Tian et al. (20) showed a significant increase in ruminal propionate, butyrate and isobutyrate levels after the addition of inulin to the rations of fattening beef cattle. Wei et al. (21) found that the concentrations of acetate and TVFA in the astragalus powder group were higher than those in the control and antibiotic groups in an *in vitro* study. Although the types and doses of additives as well as animal species were different, the test results were similar, indicating that herbal additives facilitate the degradation of feed by rumen microorganisms, promoting rapid fermentation and providing an opportunity to maintain animal metabolism. The results of this study showed that the concentrations of acetate, propionate, and TVFA in the rumen of TG1, TG2, and TG3 groups were higher than those of CG group at 2 h feeding, and the concentrations of acetate rapidly decreased 2–6 h after reaching the peak, and propionate in the experimental group began to decrease slowly after reaching the peak at 4 h feeding. It indicated that the Chinese herbal medicine SA might begin to work 2 h after the sheep ate it. Studies have found that *Ruminococcus* was the main fiber-degrading bacteria in the rumen, which could participate in the acetate metabolism, resulting in a decrease in the concentration of acetate, which promoted the rumen fermentation to tend to propionate type (22). While propionate was a glycogenic substance, the higher concentration of propionate in rumen fluid indicated the higher energy available for weight gain, which was similar to the results of our following rumen bacterial diversity. These results indicated that the addition of SA might improve the rumen fermentation function under high concentrate condition by the composition and structure of rumen microflora, change the proportion of VFA in the rumen, and further affect the digestion and absorption of dietary nutrients by the host, thereby promoting the growth of healthy animals.

### 4.2. Effect of SA added to high concentrate diets on rumen bacterial diversity in sheep

Ruminal microorganisms provide energy to ruminants by converting nutrients contained in the feed into VFA as well as microbial proteins through a series of complex biochemical reactions (23). Microbial diversity studies were conducted mainly based on conserved regions of rRNA nucleic acid sequences and bacteria were mainly based on the 16S region. Shannon and Simpson mainly reflect species diversity, while Ace and Chao measure species richness. We found that shannon, simpson indices were not significantly different between groups by rumen bacterial alpha diversity. This indicates that the rumen bacterial diversity of SA added at different doses in high concentrate diets has some similarity. Ace index and Chao index were greater than the control group in all the three experimental groups, but the difference between the groups was not significant, indicating that the species abundance was also basically the same in the four groups. In this experiment, it was found that *Ruminiclostridium* is a common genus in TG1, TG2 and TG3 groups, and *Clostridium butyricum* in *Ruminiclostridium* has the ability to improve intestinal flora and improve animal immune function and enhance disease resistance by increasing the level of humoral immunity, enhancing the intestinal mucosal barrier, and inhibiting inflammation (24, 25). This was verified by our previous findings that SA could improve immune and antioxidant functions in serum, alleviate rumen damage caused by high concentrate diets through inflammatory signaling pathway-related inflammatory factor production and release, and enhance rumen epithelial barrier function by promoting rumen epithelial tight junction protein expression in lambs (19, 26).

At the phylum level, studies have found that, after goats were injected with LPS, the number of *Firmicutes-unclassfied* and *Spirochaetes-unclassified* bacteria in the rumen was significantly increased, while the number of *Bacteroidetes-unclassfied*, *Paraprevotella* and *Centipeda* w*as* significantly decreased (27). Zened et al. (28) found that the dominant bacteria in rumen of dairy cows would not change with the change of diet, and they were still *Bacteroidetes* and *Firmicutes*. The results of this study showed that *Bacteroidetes* and *Firmicutes* were the dominant phyla in each group, and compared with the control group, the 0.5% SA group increased the abundance of rumen *Bacteroidetes* and decreased the abundance of *Firmicutes*. *Prevotella* was the most dominant species of *Bacteroidetes*, which can degrade polysaccharides such as starch, xylan and pectin in the rumen, but cannot degrade cellulose (29). It is suggested that the addition of SA to high concentrate diets enhanced the degradation of non-fibrous carbohydrates, which helped to alleviate the harm caused by excess LPS in sheep to rumen microorganisms and positively influenced the community structure of rumen microbial flora in high concentrate diets conditions. In addition, it is reported that the increase of the ratio of *Firmicutes* to *Bacteroidetes* could lead to the deposition of fat (30). In this experiment, the ratio of *Firmicutes* to *Bacteroidetes* TG1 and TG3 was lower than that of the control group, which just proves that our previous study found that SA group could reduce the concentration of triglyceride in serum (19), thus better explains that the reason for the growth promotion of SA might be that the breakdown of triglyceride provides a large amount of energy for animal growth. However, Wei et al. (21) found *in vitro* studies yet *Astragalus* group yaks had a significant decrease *Bacteroidetes* and a significant increase *Firmicutes*, which may be due to the ratio of dietary concentrate, the main active ingredients and animal breed that differ from the results of this experiment.

At the genus level, *Ruminococcus* is a kind of vatal bacteria for ruminant to utilize fiber material in rumen. In this experiment, the number of *Ruminococcus* in CG group was significantly lower than that in expermental groups, indicating that the addition of SA in the diet increased the number of rumen fiber utilization bacteria and promoted the fermentation of fiber material, which was similar to the above-mentioned change rule of pH value in rumen. At the same time, we also demonstrated by qPCR results that the number of *Ruminococcus* albicans in experimental groups increased significantly compared with the control group. However, Zhou et al. (31) found that the relative abundance of *Ruminococcus* was positively correlated with the release of IL-1β in the body, which might increase the risk of organ and tissue damage. Our previous experimental results found that the difference of IL-1β in serum between the experimental group and the control group was not significant, which may be caused by the difference of animal species and detection sites, and its specific mechanism needs further verification. Liu et al. (32) found that *Biochanin* A significantly increased the abundance of the dominant rumen bacterium *Prevotella ruminicola* protein hydrolysing bacteria. *P. ruminicola* synthesizes proteases and deaminases to generate ammonia from dietary protein for using by bodies. Emma et al. (33) found that *Clostridiaceae* showed a high positive correlation with crude protein content, protein digestibility, and total energy, and a weaker positive correlation with fat digestibility. The percentage of *Clostridiaceae* was significantly higher in TG1 and TG2 groups in this experiment, indicating that the addition of 0.1–0.3% SA to the diets could improve the number of protein-using bacteria, thus ensuring better nutrient absorption in lambs under high concentrate conditions.

### 4.3. Effect of SA added to high concentrate diets on rumen fungi diversity in sheep

Anaerobic fungi that colonize the digestive tract of ruminants produce and secrete a series of polysaccharide-degrading enzymes, including glucoside hydrolases, cellulases and xylanases, which can better facilitate the utilization of dietary cellulose by the rumen (34). Most studies have shown that feeding practices, feed concentrate-to-forage ratio and diet nutrient levels can affect the bacterial diversity and composition of the dominant flora in the rumen (35, 36), while there have fewer reports on rumen fungi in sheep. Ye et al. (37) found that the addition of fructans to the ration could affect the Ace, Shannon and Simpson index of rumen fungi in Holstein cows, but did not significantly affect the overall diversity and abundance of rumen fungal flora. We found that Simpson showed TG1 > TG2 > TG3 > CG among the groups by sheep rumen fungal Alpha diversity, and Sobs, Shanon, Ace and Chao index control groups were all slightly higher than the three experimental groups, but none of the differences were significant (*p* > 0.05), which was similar to the results of Wang et al. (38) suggesting that the addition of SA in high-concentrate diet had different degrees of sheep rumen fungal diversity and abundance, but they also had certain similarities. In this experiment, the rumen fungal composition from sheep rumen was found to be more abundant in the TG1 group both at the phylum level and genus level, and the fungal composition at the rumen genus level in this experiment revealed that *Sarocladium* was a genus unique to the TG1, TG2, and TG3 groups, and *Sarocladium* can degrade cellulose and xylan (39), which also further indicates that the addition of SA to high concentrate diets might enrich the species of fibrous degrading bacteria.

At the phylum level, Li et al. (40) found that the dominant phyla of rumen fungi in the diets of small-tailed Han sheep consuming diets with at different protein levels was mainly *Ascomycota*, *Basidiomycota* and *Neocallimastigomycota*. Han (41) found in the study of Shaanxi cashmere goats that the dominant phylum of rumen fungi in different concentrate-to-forage ratio diets groups was *Ascomycota*, and it was mainly involved in the degradation of recalcitrant nutrients in the feed. In contrast, Peng et al. (42) added grape seeds to the sheep diet and found that the dominant rumen fungi of Dorang sheep were *Neocallimastigomycota* and *Ascomycota*, which were consistent with the experimental results. It follows that different species and feeding practices, as well as differences in the main active substances could alter the relative abundance of the dominant rumen phylum. *Neocallimastigomycota* was a functional fungus widely present in the digestive tract of herbivorous ruminants, and played an important role in degrading lignified cellulose (43), and could also provide the host with nutrients required for vital activities by consuming rumen degradable proteins (44). The relative abundance of *Neocallimastigomycota* in all three experimental groups in this experiment was numerically higher than that of the control group, and the highest in the TG1 group, indicating that the addition of appropriate amounts of SA to high concentrate diets would enhance cellulose degradation and provide energy for animal metabolism. In this experiment, the relative abundance of *Neocallimastigomycota* was numerically higher in all three test groups than in the control group, and was highest in the TG1 group, indicating that the addition of SA to high concentrate diets would enhances the degradation of cellulose and provides energy for animal metabolism.

At the genus level, Wang et al. (45) showed that *Piromyces* fungal inoculants increased the *in vitro* digestibility of dry matter and neutral detergent fiber of silage after 30 days of fermentation. *Piromyces*, *Unclassified*-Neocallimastigaceae and *Neocallimastix* belong to *Neocallimastigomycota*, which had the ability to digest cellulose efficiently. The results of this experiment showed a relatively high level of these three fungal genera, suggesting that the addition of SA in high concentrate diets would improve the utilization of cellulose, which was also verified in the above results of rumen bacterial diversity. Dietary concentrate-to-forage ratio has a large effect on the characteristics of rumen fungi. Yang et al. (46) found that feeding a high concentrate full-price pelleted diet inhibited the activity of anaerobic fungi and rumen fiber-degrading bacteria in calves. Zhu et al. (47) reported that concentrates inhibited the production of fungal xylan isomerase, which might be related to the induction of crude fiber-like substrates, while *Piromyces* can produce xylan isomerase and increase the number of cellulose degrading bacteria (48, 49). In this experiment, the relative abundance of *Piromyces* in TG1 group was much higher in value than the control group, suggesting that the addition of SA to high concentrate diets might increase the activity of xylan isomerase and promote the proliferation of fiber-degrading bacteria in the rumen of sheep, which helped maintain the ruminal microecological balance of lambs under high concentrate conditions.

## 5. Conclusion

In this study, the addition of SA to the high grain diet could increase the concentration of acetate, propionate, butyrate, TVFA and pH in the rumen of sheep, and reduce the concentration of NH_3_-N in the rumen. The dominant rumen bacteria after adding SA to the high grain diet were *Bacteroidetes* and *Firmicutes*, which increased the relative abundance of *Ruminococcus*, *Phocaeicola* and *Clostridiales* in the rumen of SA group. And the dominant phylum of sheep rumen fungi was *Neocalymastigomycota*, and the dominant genus was *unclassified-Neocalymastigaceae*, which increased the relative abundance of rumen *Neocalymastigomycota* and *Piromyces* in the 0.1% SA group to a certain extent. This has a positive effect on promoting rumen fermentation and fine-tuning the rumen microbial ecosystem to improve sheep health.

## Data availability statement

The datasets presented in this study can be found in online repositories. The names of the repository/repositories and accession number(s) can be found at: https://www.ncbi.nlm.nih.gov/bioproject; PRJNA963087.

## Ethics statement

The animal study was reviewed and approved by Inner Mongolia Agricultural University Institutional Animal Care and Use Committee. Written informed consent was obtained from the owners for the participation of their animals in this study.

## Author contributions

All authors participated in study design, data acquisition and analysis, and preparation of the manuscript.

## Funding

This work was financially supported by National Natural Science Foundation of China (31860658).

## Conflict of interest

The authors declare that the research was conducted in the absence of any commercial or financial relationships that could be construed as a potential conflict of interest.

## Publisher’s note

All claims expressed in this article are solely those of the authors and do not necessarily represent those of their affiliated organizations, or those of the publisher, the editors and the reviewers. Any product that may be evaluated in this article, or claim that may be made by its manufacturer, is not guaranteed or endorsed by the publisher.
